# Towards highest peak intensities for ultra-short MeV-range ion bunches

**DOI:** 10.1038/srep12459

**Published:** 2015-07-27

**Authors:** Simon Busold, Dennis Schumacher, Christian Brabetz, Diana Jahn, Florian Kroll, Oliver Deppert, Ulrich Schramm, Thomas E. Cowan, Abel Blažević, Vincent Bagnoud, Markus Roth

**Affiliations:** 1 GSI Helmholtzzentrum für Schwerionenforschung, Planckstr. 1, D-64291 Darmstadt, Germany; 2 Helmholtz Institut Jena, Helmholtzweg 4, D-07734 Jena, Germany; 3 Technische Universität Darmstadt, Institut für Kernphysik, Schloßgartenstraße 9, D-64289 Darmstadt, Germany; 4 Helmholtz-Zentrum Dresden-Rossendorf, Bautzner Landstr. 400, D-01328 Dresden, Germany; 5 Technische Universität Dresden, D-01062 Dresden, Germany

## Abstract

A laser-driven, multi-MeV-range ion beamline has been installed at the GSI Helmholtz center for heavy ion research. The high-power laser PHELIX drives the very short (picosecond) ion acceleration on *μ*m scale, with energies ranging up to 28.4 MeV for protons in a continuous spectrum. The necessary beam shaping behind the source is accomplished by applying magnetic ion lenses like solenoids and quadrupoles and a radiofrequency cavity. Based on the unique beam properties from the laser-driven source, high-current single bunches could be produced and characterized in a recent experiment: At a central energy of 7.8 MeV, up to 5 × 10^8^ protons could be re-focused in time to a FWHM bunch length of *τ* = (462 ± 40) ps via phase focusing. The bunches show a moderate energy spread between 10% and 15% (ΔE/E_0_ at FWHM) and are available at 6 m distance to the source und thus separated from the harsh laser-matter interaction environment. These successful experiments represent the basis for developing novel laser-driven ion beamlines and accessing highest peak intensities for ultra-short MeV-range ion bunches.

Laser-based ion acceleration as a source for intense, MeV-range ion bunches is discussed for many possible applications: in the context of fusion science^1^, the creation of warm dense matter^2^,^3^,^4^ or as diagnostic tool^5^,^6^,^7^ as well as for medical applications^8^,^9^. A well understood and widely-used mechanism for laser-based ion acceleration is the TNSA (target normal sheath acceleration^10^,^11^). Typically accelerated protons show excellent beam properties with respect to bunch intensity and emittance^12^ and also the feasibility of efficiently accelerating heavier ions could be demonstrated experimentally^13^. However, the beam suffers from a large divergence and continuous broad energy spectrum, while for most applications a collimated bunch with defined energy spread is necessary. First promising results in beam shaping could be achieved via the application of pulsed solenoids^14^,^15^, permanent magnetic quadrupoles^16^,^17^ or laser-triggered microlenses^18^.

For the also necessary manipulation of the longitudinal bunch dynamics, injection into a synchronous radiofrequency (rf) field yields high potential and a first conceptual demonstration was performed in Japan^19^,^17^. As interest in such novel beamline concepts arises^20^,^21^, the German national collaboration LIGHT (Laser Ion Generation, Handling and Transport^22^), has built a test beamline at the GSI Helmholtz center for heavy ion research as the central part of the collaboration’s agenda. This beamline exploits the TNSA mechanism to provide a very compact proton source with energies currently reaching up to 28.4 MeV. The acceleration is driven by GSI’s PHELIX laser (Petawatt High Energy Laser for Ion eXperiments^23^), which is focused at a laser intensity of 5 × 10^19^ W/cm^2^ onto a thin metal foil target (typically 5 or 10 *μ*m thin gold or titanium foils). From the continuous and highly divergent source spectrum a specific energy can be selected and collimated via a pulsed high-field solenoid, which can be operated at up to 9T field strength. The obtained source parameters are in the typical range for TNSA experiments within the given laser and target parameters and it is possible to accelerate more than 10^12^ protons above 4 MeV energy in total with about 10^10^ protons in a 1 MeV energy bin around 10 or 8 MeV. Via chromatic focusing with the pulsed solenoid, up to one third of the protons in such an energy bin can be captured and transported through the beamline within a collimated bunch with still relatively large energy spread (about 20% FWHM). Although thus the overall capture efficiency is on the sub-percent level, still large single-bunch particle numbers above 10^9^ can be created. However, the beamline is routinely not operated at its limit and also typical shot-to-shot fluctuations of up to a factor of 2 in particle numbers are observed. This first step of the experiment is described in detail in^24^ and an illustration given in Fig. 1.

The next step has been the longitudinal phase rotation of the bunch via applied electrical fields within a rf cavity, running at 108.4 MHz and providing a total electrical potential of more than ±1 MV. Injection of the bunch at a synchronous phase of Φ_*S*_ = −90 deg leads to a rotation around the central energy in longitudinal phase space and at a certain rf input power to an efficient energy compression of the bunch; less than 3% energy spread could be achieved in a previous experimental run^25^ for protons at 9.4 MeV energy and particle numbers larger than 10^9^.

With increasing rf power the bunch can also be ‘over-rotated’ in phase space, leading to a situation of a well-ordered energy distribution within the bunch with the slower particles at the front and the faster particles at the back. Along a further propagation length, the faster will catch up with the slower particles and at one specific distance a minimum in the bunch length will be reached. The mechanism is called *phase focusing* and is illustrated in Fig. 2 together with the alternative operation mode for *energy compression*. While the latter was already demonstrated in a previous run in 2013, finally the phase focusing could be experimentally accomplished recently. This completes the initial commissioning phase of this novel laser-driven ion beamline, available now at GSI and representing the focus of this paper.

The comparative simulations are performed with the TraceWin code from *cea*^26^ and use beam parameters, that are adapted to the experimental findings to most precisely model the experiment. The specific parameters used here will be discussed later in context with the experimental results.

## Setup and Diagnostics

The mechanism of phase rotation for temporal bunch compression essentially relies on the quite large energy spread of the bunch. Therefore the bunch will quickly defocus again in phase behind the specific focal position. Our simulations predict this focal position to be at 3.45 m behind the cavity (6 m to source) for protons of 7.8 MeV energy and a total applied gap voltage of 0.96 MV. This position was chosen as diagnostic position in the performed experiments and the rf power varied to scan the bunch length at this specific position.

The first part of the experimental setup is the same as described in^25^: The pulsed solenoid is placed 80 mm behind the laser matter interaction point and collimates a specific proton energy via chromatic focusing (the solenoid field strength can be assumed constant for the proton transition time). A drift leads then to the 550 mm long rf cavity, starting at 2 m to the source and consisting of three acceleration gaps. Behind the rf cavity the beamline is extended up to a diagnostic chamber at 6 m distance to the source: Two permanent magnetic quadrupole doublets (QD) keep the beam transversally confined along the drift. They are placed at 3.2 m and 5 m distance to the source and consist of 50 mm long, 25 T/m strong permanent magnetic quadrupoles in a Hallbach design. Optionally, for a steeper final focusing a third QD can be inserted, consisting of a (80 mm, 85 T/m) and a (45 mm, 105 T/m) permanent magnetic quadrupole in Hallbach design. A schematic of the full beamline is shown in Fig. 3.

Adjustment and online control of source and machine parameters are available: The current through the pulsed solenoid and the phase and relative voltage of the rf wave within the cavity are monitored on-shot as well as the PHELIX laser parameters, including a high-precision relative timing measurement for the synchronization of laser and rf. Concerning the accelerated proton bunch, the beamline has several possible diagnostic ports for characterization: Transverse beam profile, spectral characteristics and proton numbers can be obtained with dosimetry films in stacked configuration (Radiochromic Imaging Spectroscopy^27^), and the temporal bunch profile measurement is performed with two complementary methods:

On the one hand, the bunch hits a large, fast plastic scintillator (BC-422Q from Saint Gobain, 1% benzene quenching, decay time *τ* = 0.7 ns). Besides picturing the full scintillator area and thus recording the transverse beam profile with a fast dicam pro (from *pco*), a horizontal lineout at the center of the scintillator is recorded with a streak camera (for visible light, from *Hamamatsu*), using a 50 ns streak time and a resulting temporal resolution of Δ*τ* = ±0.2 ns. On the other hand, the scintillator has a 1.5 mm diameter central hole to let part of the beam pass through and hit a specially designed fast diamond detector, which consists of a 13 *μ*m thin pcCVD (polycrystalline chemical vapor deposition) diamond plate with an active detection area of 0.8 mm^2^. An applied field gradient of 2.3 V/*μ*m is used to quickly drain the free charges, that are created by the protons (electron-hole pairs) while passing the detector. A special impedance matching results in a calculated signal rise time of the detector of only *τ* = RC ≈ 40 ps. This detector has been developed in collaboration with GSI’s detector laboratory and reaches the necessary time resolution. It is connected to a 8 GHz oscilloscope using a minimized cable length for signal transport of less than 0.5 m high-frequency compatible SMA cables.

The arrangement in the diagnostic chamber is illustrated in Fig. 4 and includes relevant dimensions and distances. As the dosimetry measurements with radiochromic film (RCF) could not be done in parallel to the other measurements, particle numbers have not been recorded routinely. However, they could be determined to be at a level of 3 × 10^8^ and constant within the typical shot-to-shot fluctuations from the source (±50%) as observed in previous campaigns^24^,^25^.

### Bunch characterization

For reference purposes the transported proton bunch was first characterized at the diagnostic position at 6 m behind the source without the rf cavity running. The solenoid was always driven at a peak current of 7.8 kA, resulting in an maximum magnetic field of 6.55 T. This leads via energy selection through chromatic focusing to a central bunch energy of E_0_ = 7.8 MeV protons. The central part of the bunch is well fitted by a Gaussian with (21 ± 3)% energy spread (ΔE/E_0_ at FWHM), which is also in good agreement with the results from previous campaigns^24^,^25^. Due to expected additional losses in the new transport section the measured particle numbers were slightly lower and in the range of 1.5 × 10^8^ to 5 × 10^8^ protons within FWHM. These values are obtained from the dosimetry measurements and served as input parameters for the comparative simulation studies.

Switching on the rf in the cavity, first an absolute calibration of the synchronous phase Φ_*S*_ is necessary to synchronize the laser and the rf and being able to inject the bunch at the correct phase of Φ_*S*_ = −90 deg. This was done by scanning the rf phase and diagnosing the bunch with the streak camera and optionally a dipole spectrometer (as described in^25^). After this absolute calibration of the timing system, the synchronous phase can be adjusted in advance to a precision of ΔΦ_*S*_ = ±12 deg and measured on-shot with even ΔΦ_*S*_ = ±2 deg, which defines the relative uncertainty for all given values for Φ_*S*_ in this paper.

### Phase focusing

**Figure 2:**
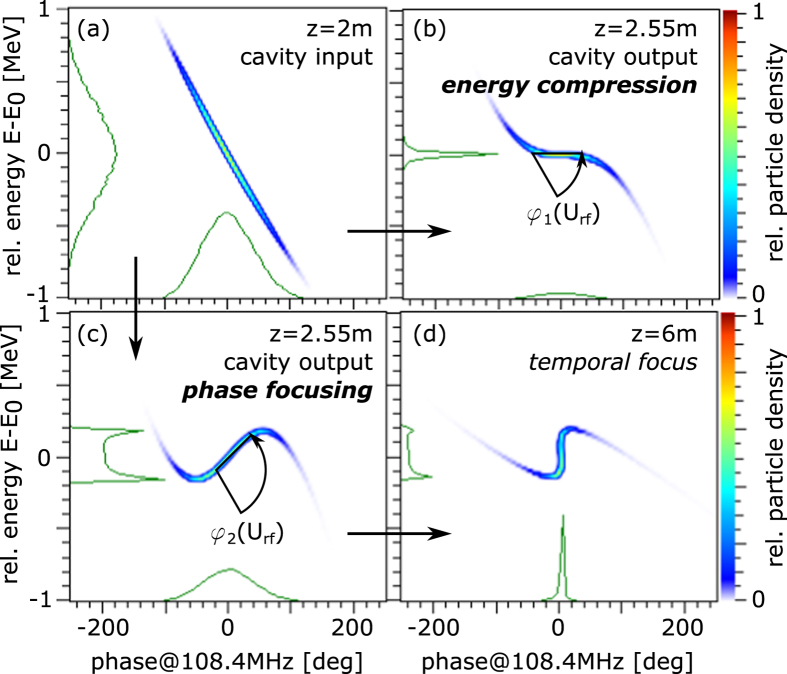
Illustration of phase rotation in longitudinal phase space via applied rf. The input beam is shown for reference in (**a**) and the two operational modes of interest are depicted in (**b**) and (**c**): At an injection phase of Φ_*S*_ = −90 deg the bunch is rotated around the central energy by an angle *φ* in dependency on the rf amplitude U_*rf*_. (**b**) represents the *energy compression mode*, see^25^ and (**c**) the *phase focusing mode*, which is described in this paper and leads to (**d**) a focus of the bunch in the time domain after a certain drift length.

**Figure 3:**
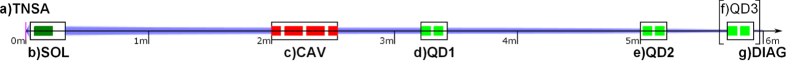
The setup of the current LIGHT beamline at GSI. (**a**) The PHELIX laser drives the acceleration of a broad proton spectrum up to 28.4 MeV energies via the TNSA mechanism. (**b**) A specific energy can be selected and collimated by a pulsed high-field solenoid, in this case 7.8 MeV protons, and the bunch is (**c**) rotated in longitudinal phase space within a rf cavity. Two quadrupole doublets, (**d**) and (**e**), provide for the beam transport up to (**g**), a diagnostic chamber at 6 m distance to the source. (**f**) represents an optionally third quadrupole doublet for a steeper final focusing of the bunch.

**Figure 4:**
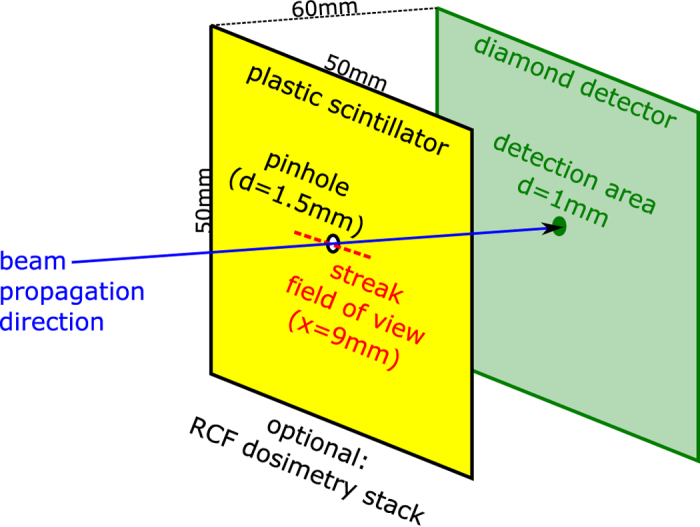
Setup of the beam diagnostics . The bunch hits the fast plastic scintillator at 6 m distance to the TNSA proton source. The full transverse profile of the scintillator is recorded with a camera and a horizontal lineout is imaged to a streak camera. Through a free, centered aperture within the scintillator a part of the beam passes towards the diamond detector for a time-of-flight measurement. Optional to the scintillator, a dosimetry measurement is possible with a stack of radiochromic films.

For the phase focusing experiments, the bunch is injected into the rf field at Φ_*S*_ ≈ −90 deg synchronous phase, thus efficiently rotated in longitudinal phase space as pictured in Fig. 2. The bunch length is recorded at the detection position at 6 m behind the source with a streak and a diamond detector and the rf amplitude is varied to find the minimum achievable bunch length. The rf amplitude cannot be measured directly, but is determined by the (known) rf input power and the shunt impedance of the cavity. A comparison to the expected values from the simulations will be done later on and instead an (arbitrarily) normalized rf amplitude U_*r,f,n*_ will be given as the experimental observative, which is directly proportional to the real amplitude.

**Figure 5:**
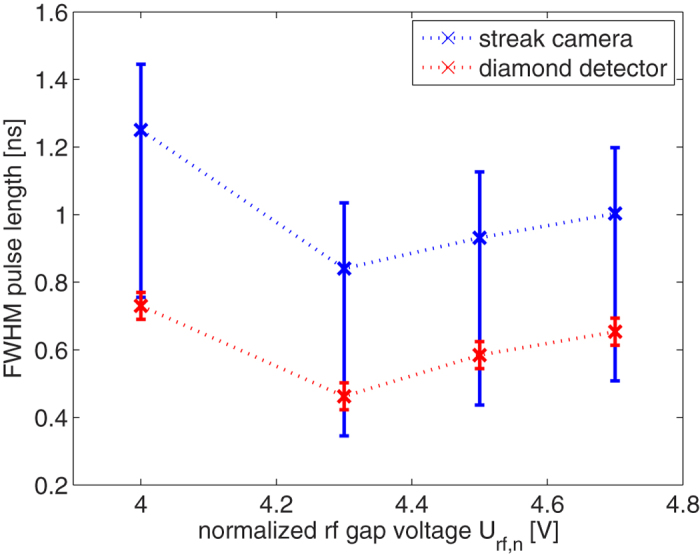
Measured FWHM bunch lengths within the diagnostic chamber at an injection phase into the rf cavity of Φ_*S*_ = −90 deg and varying rf amplitude U_*r,f,n*_. The streak data gives an upper boundary only due to the long decay time of the scintillator, while the diamond detector provides a much better time resolution. With respect to the general behavior, both detectors are in very good agreement.

**Figure 6:**
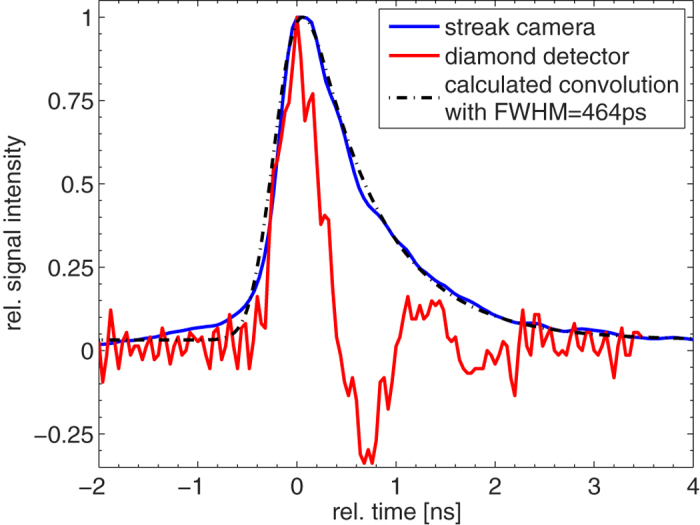
Temporal profile of the shortest obtained bunch with *τ *= (462 ± 40) ps (FWHM), measured by the streak and diamond detector in parallel. Both signals are normalized to maximum value and furthermore this maximum value is shifted to time t = 0. Also indicated: The convolution of a Gaussian signal (FWHM = 462 ps) with an exponential decay function (*τ *= 700 ps), which reproduces the scintillator signal perfectly.

Scanning the rf power around the value of optimum temporal bunch compression for the given setup reveals the expected minimum as shown in Fig. 5. Both, diamond and streak detector show a consistent behavior. A minimum FWHM pulse length of *τ* = (462 ± 40) ps is measured with the diamond detector. The streak camera in this case is only able to set the upper limit to the pulse length, as it suffers from two major error contributions: a symmetric error through the finite entrance slit width (Δ*τ* = ±200 ps) and an asymmetric error through the still large signal decay time of the scintillator (*τ*_*decay*_ = 0.7 ns according to manufacturer’s specification). A direct comparison of the response of both detectors is given in Fig. 6, showing the measured signal at the optimum temporal compression parameters. While both show a rapid signal rise time, the signal decay is dominated for the streak camera by the slow scintillator decay time and the diamond shows an undershoot oscillation due to intrinsic detector characteristics. Calculating the convolution of a Gaussian with a FWHM of 462 ps and the exponential decay function of the scintillator reproduces the measured signal very good and thus the measurement of the diamond detector can be verified with this complementary measurement via the streak camera. (The convolution pictured in Fig. 6 has an additional slight shift upwards to match the non-zero offset of the experimental data.)

Similarly, the undershoot oscillation of the diamond detector can be identified as a detector intrinsic: For once, an in reality occuring second particle peak 1 ns after the main bunch would be visible in the streak detector, too. Moreover, the oscillating behavior could be identified as an artefact of a resonant circuit within the detector electronics.

## Discussion

The 7.8 MeV proton bunch could be temporally re-compressed to less than 500 ps length. Still, this is far more than the original bunch length (approximately 1 ps acceleration time). First, a major reason is the difference in the propagation path for all the particles within the bunch. The pulsed solenoid collects particles from a large solid angle (±100 mrad) and the covered propagation length while passing through the solenoid is quite different for e.g. a proton passing the solenoid straight on axis or a proton entering at 100 mrad, which is then bent back towards the axis on a spiral trajectory. Further contributions of the same kind are added along the beamline within the different elements and results in a temporal broadening for particles with the exact same energy. These effects are included in the comparative simulation studies, which predict a minimum FWHM pulse length for a proton bunch with a central energy of 7.8 MeV of Δ*τ*_Δ*S*_ ≈ 70 ps just due to the difference in propagation paths.

Secondly, the temporal focus is very sensitive to the experimental parameters (rf phase, rf amplutide and detection position). The practically limited adjustment accuracy needs to be taken into account here: the synchronization jitter of ±12 deg adds to the total error and especially an increased step size for the rf power scan might reveal a slightly shifted position of the minimum. A more detailed scan is planned for future experiments.

### Summary and Outlook

In summary, a worldwide unique laser-driven beamline is now available at GSI, providing highest proton currents due to the unique source parameters via phase focusing. In recent experiments, up to 5 × 10^8^ (±20%) protons could be compressed in time to a bunch length of *τ* = (462 ± 40) ps, thus to a peak particle current of 170 mA. The transverse final focusing was not yet optimized and the minimum transverse beam size at the longitudinal focus position was measured to 3 × 18 mm^2^.

As the next steps in the further development of the LIGHT beamline, an upgrade of the final focusing system is planned to minimize the transverse beam profile and thus access highest bunch intensities. Also the ion species will be varied so that acceleration and beam shaping of carbon and flourine can be explored. The possibility of efficient acceleration of these ion species has already been demonstrated within the TNSA regime^13^. With these forseen upgrades, the beamline might enter a comparable parameter regime as the proposed NDCX-II machine^28^.

Finally, the beamline profits from the unique experimental possibilities at its location at GSI: Also available for combined experiments are the conventional ion beam from the UNILAC accelerator and the high energy laser *nhelix*^29^ as well as multifold ion beam and plasma diagnostics.

## Additional Information

**How to cite this article**: Busold, S. *et al.* Towards highest peak intensities for ultra-short MeV-range ion bunches. *Sci. Rep.* **5**, 12459; doi: 10.1038/srep12459 (2015).

## Figures and Tables

**Figure 1:**
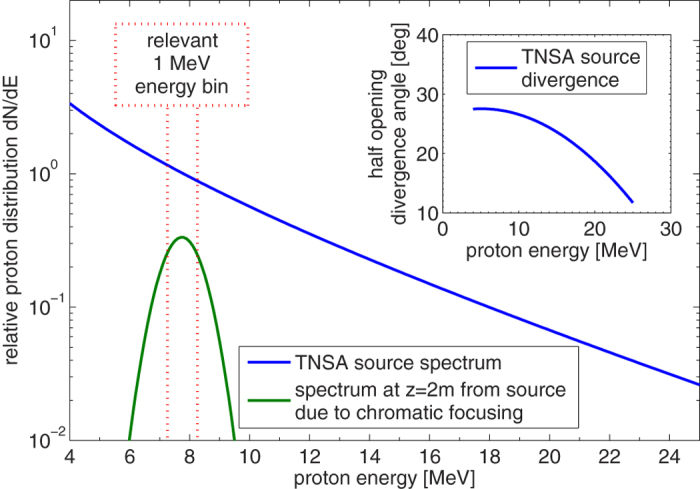
Shown are the accesssible source parameters via TNSA (energy-dependent proton distribution function and half opneing divergence angle) at the used experimental area Z6 at GSI Darmstadt. Also indicated the effect of spectral filtering via chromatic focusing, in this case with the solenoid adjusted to collimate protons with an energy of 7.8 MeV.
